# Prediction of Molecular Properties Using Molecular Topographic Map

**DOI:** 10.3390/molecules26154475

**Published:** 2021-07-24

**Authors:** Atsushi Yoshimori

**Affiliations:** Institute for Theoretical Medicine, Inc., 26-1, Muraoka-Higashi 2-chome, Fujisawa 251-0012, Japan; yoshimori@itmol.com; Tel.: +81-466-52-8317

**Keywords:** generative topographic mapping, convolutional neural network, property prediction, data augmentation

## Abstract

Prediction of molecular properties plays a critical role towards rational drug design. In this study, the Molecular Topographic Map (MTM) is proposed, which is a two-dimensional (2D) map that can be used to represent a molecule. An MTM is generated from the atomic features set of a molecule using generative topographic mapping and is then used as input data for analyzing structure-property/activity relationships. In the visualization and classification of 20 amino acids, differences of the amino acids can be visually confirmed from and revealed by hierarchical clustering with a similarity matrix of their MTMs. The prediction of molecular properties was performed on the basis of convolutional neural networks using MTMs as input data. The performance of the predictive models using MTM was found to be equal to or better than that using Morgan fingerprint or MACCS keys. Furthermore, data augmentation of MTMs using mixup has improved the prediction performance. Since molecules converted to MTMs can be treated like 2D images, they can be easily used with existing neural networks for image recognition and related technologies. MTM can be effectively utilized to predict molecular properties of small molecules to aid drug discovery research.

## 1. Introduction

Evaluation of molecular property has a pivotal role in process of drug discovery [1]. Although experimental methods such as in vitro and in vivo tests are both time and cost consuming, they have been widely used across the process [2]. The prediction of molecular properties on the bases of molecular structures is one of the most classical chemoinformatics tasks and provides alternative approaches to profile molecules efficiently [3]. Lombardo et al. demonstrated that prediction of absorption, distribution, metabolism, excretion, and pharmacokinetics (ADME-PK) is an integral part of the current industrial drug discovery paradigm [4]. A variety of property predictive models based on machine learning (ML) approaches random forest [5], support vector machine [6], XGboost [7], LightGBM [8], and others [9,10] have been proposed. In the models, descriptors and/or fingerprints are used as molecular representation. The selection of molecular descriptors determines the quality of the predictive model [11]. Stahura et al. proposed an entropy-based descriptor selection method in the prediction of aqueous solubility of organic molecules [12]. The predictive model constructed using selected descriptors showed consistent high prediction accuracy in binary QSAR calculations. Awale et al. performed ADME property prediction by using matched molecular series analysis (MMSA) [13]. The prediction accuracy was comparable to a standard ML model. However, because MMSA predicts the property of a new compound based on relationship between experimental values of similar MMS pair, the source of the predictions can be exactly traced back. In recent years, deep learning (DL) methods have received a lot of attention as methods to accomplish the classical chemoinformatics tasks due to their capacity to learn intricate relationships between structures and properties [14,15,16,17,18,19] and capability of feature selection [20]. In molecular property prediction, two major DL models, descriptor-based models and graph-based models, have been proposed [21,22,23,24,25]. In descriptor-based models, descriptors and/or fingerprints are used as input to the models [22]. In graph-based models such as graph neural networks (GNNs), a molecular graph is used to represent a molecule by considering the atoms as vertices and the bonds as edges of a graph. The molecular graph is then used as input to the models [23,24,25]. Wang et al. proposed composite model combining molecular fingerprint and molecular graph [26]. The model is accurate and achieves the best results on some open benchmark datasets. In other approaches using the DL model, the current successful models in natural language modeling have been used to predict molecular property [27,28]. In the models, molecules are represented as strings by Simplified Molecular-Input Line-Entry system (SMILES) [29]. Wang et al. proposed SMILES-BERT [27], which uses the recent natural language modeling work BERT [30], to predict molecular property. To evaluate its predictive performance, they used three datasets including LogP, PM2, and PCBA-686978 [27]. On the other hand, as inputs to deep learning, a simpler and more intuitive molecular representation is by two-dimensional (2D) molecular images which were combined with convolutional neural networks (CNNs) to predict molecular properties or activities [31,32,33,34]. Zhong et al. used a pre-trained DenseNet121 [35]—the state-of-the-art architecture of CNNs developed in 2016—for transfer learning to construct a predictive model of contaminant reactivity toward OH radicals [34]. By using molecular images as input, it is a great advantage to be able to use existing well-trained neural networks in the field of image recognition. Although molecular images are effective input data for CNNs, it is necessary to prepare multiple images from a single molecule because molecules can be represented by multiple types of images [33,34]. Here, we developed Molecular Topographic Map (MTM), which represents a molecule as a 2D matrix data (2D image). The MTM is generated from the atomic features set of a molecule using Generative Topographic Mapping (GTM) [36,37]. In this method, one MTM is generated from one molecular structure just like in molecular fingerprint generation. The performance of molecular property prediction based on MTM was equal to or better than that based on Morgan fingerprint (Morgan FP) [38,39,40] or MACCS keys [41]. Furthermore, data augmentation of MTMs using mixup [42] was found to be effective in improving the prediction performance.

## 2. Results

In the first section, a procedure for the generation of atomic features set from a molecule is described. The second section discussed the generation of MTM from the atomic features set. Additional methodological and computational details are provided in the Methods section. Subsequent sections report visualization and similarity matrix analysis of amino acids and property predictions using MTMs as input.

### 2.1. Atomic Features Set

**Figure 1:**
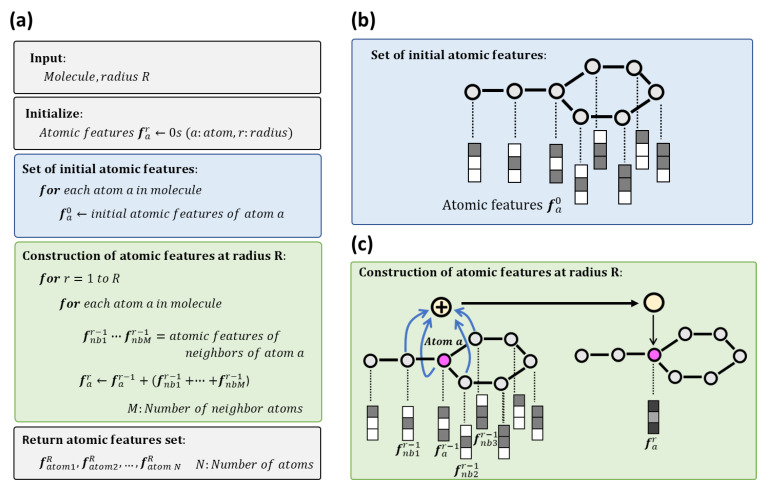
Generation of atomic features set. (**a**) Pseudocode of atomic features set. (**b**) A visual representation of the set of initial atomic features. (**c**) The construction of atomic features at radius R.

The procedure of generating atomic features set, which is illustrated in Figure 1, is based on the algorithms of Morgan FP (circular fingerprint) [38,39,40] and neural graph fingerprint [24]. Morgan FP is a method of encoding the structure of a molecule. In the process of generating Morgan FP, the environment of each atom is stored into several circular layers up to a specific radius. Neural graph fingerprint is an extension of Morgan FP to operate directly on graphs (molecular structure) in neural networks. As illustrated in Figure 1a, to generate the atomic features set, molecular structure and radius R are needed as input data. Initial atomic features are represented by vector of 0 s and 1 s indicating the existence of a specific atomic feature. After the initialization, the vectors are set to each atom (Figure 1a,b). The atomic features used in the initial assignment to atoms are shown in Table 1. In subsequent steps, the atomic features of each atom are summed up with that of its neighbor atoms. This step is repeated R times, specified as the radius (Figure 1a,c). As a result, the same number of atomic features as the number of atoms in a molecule can be obtained. The multiple atomic features obtained here is referred to as atomic features set.

### 2.2. Molecular Topographic Map

**Figure 2:**
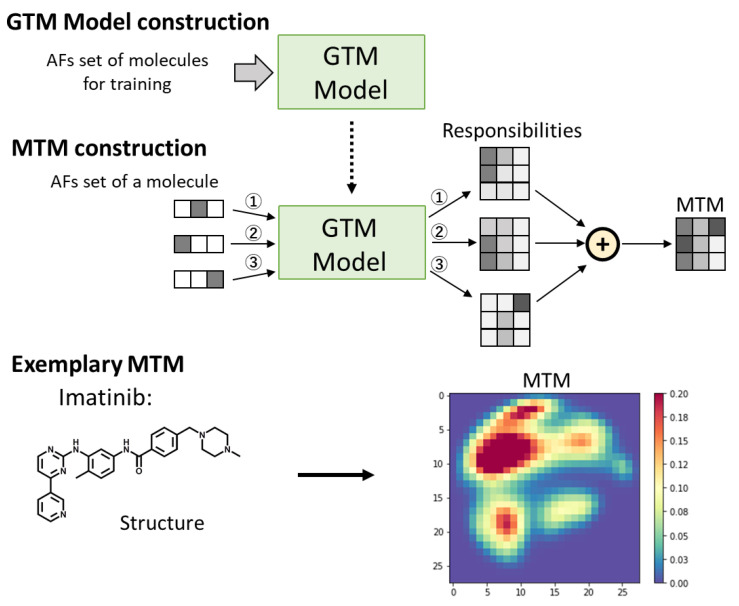
The workflow of MTM generation. GTM model is constructed using the Atomic Features (AFs) set of molecules for training. The GTM model is used for generating MTM from the AFs set of a molecule. The MTM of imatinib is shown as an exemplary MTM.

An MTM represents a molecule as a 2D matrix data. The data can be visualized as a heatmap like 2D image. In order to generate MTMs, a GTM model is constructed using the atomic features set obtained from multiple molecules for training (Figure 2, upper). Before the training, duplicated atomic features are removed from the atomic features set. GTM is a nonlinear latent variable model, which enables mapping of high dimensional data to a two-dimensional space. After the GTM construction, the multiple atomic features of a molecule are mapped onto 2D space using the GTM model. The 2D space is represented by points on a regular grid. The probability of the atomic features to be on the regular grid is calculated. The probabilities are obtained from the GTM model as “responsibilities”.

The MTM is generated by summing up the responsibilities obtained from the atomic features set of a molecule (Figure 2, middle). As an exemplary MTM, the MTM of imatinib is shown in Figure 2 (lower), which is represented by a regular grid of size 28 by 28 as a heatmap. High-value data points (red points) on the heatmap mean regions of high aggregation of similar atomic features, whereas low-value data points (blue points) mean otherwise. Similar to molecular fingerprint generation, in this method, one MTM is generated from one molecular structure.

### 2.3. Molecular Topographic Maps of 20 Amino Acids

**Figure 3:**
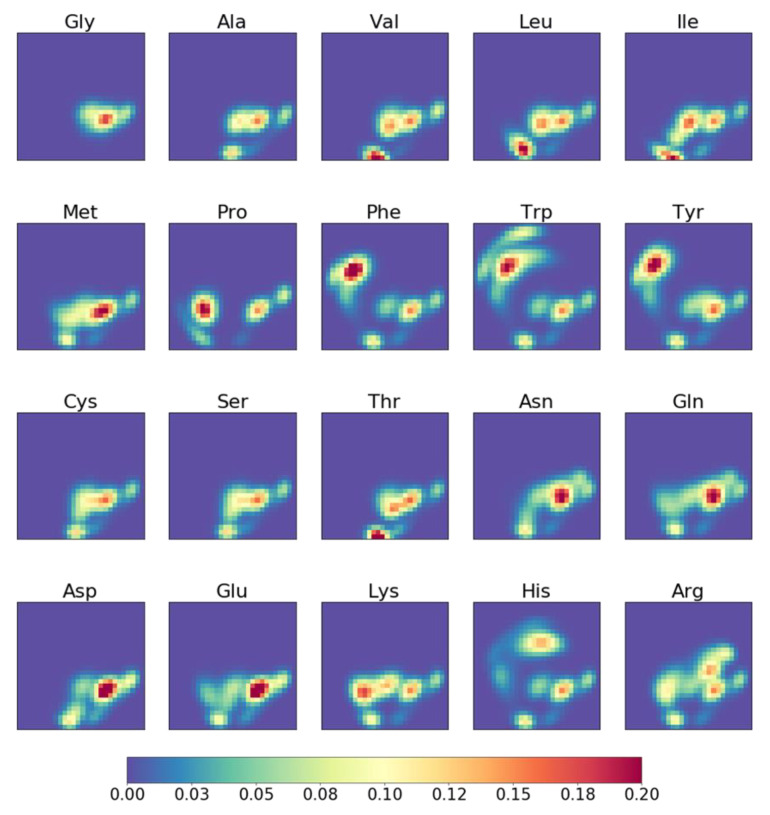
MTMs of 20 amino acids.

**Figure 4:**
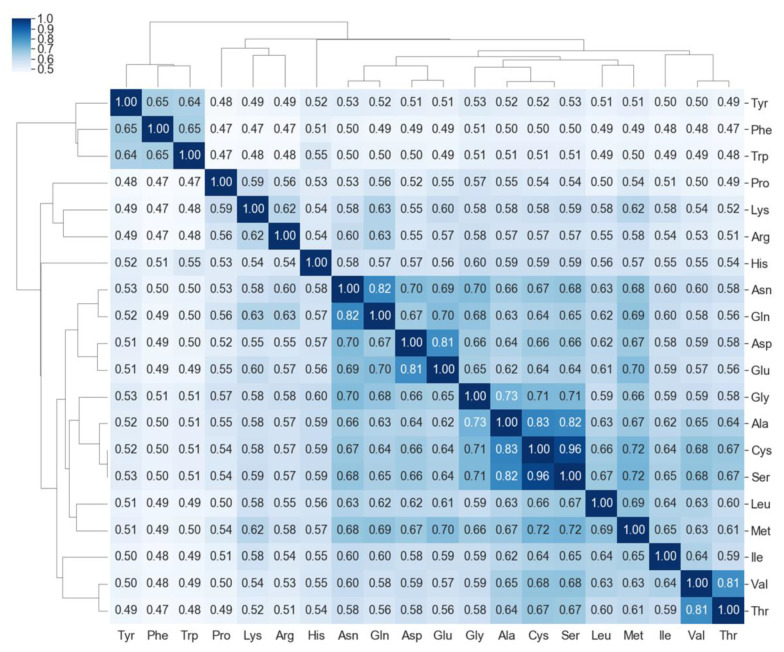
Clustered similarity matrix of MTMs of 20 amino acids.

Figure 3 shows MTMs of 20 amino acids, whereby each MTM is represented by a heatmap of size 28 × 28. Based on visual inspection, there are apparent differences between the MTMs, reflecting differences in the structures of amino acids. For example, MTMs of small-sized amino acids, such as Gly, Ala, Val, Cys, and Ser, have small regions of high-value data points, and Gly has the smallest of such region. There are very similar MTM pairs, such as Cys/Ser, Val/Thr, Asn/Gln, and Asp/Glu, and each pair of amino acids exhibit similar structures. Amino acids with aromatic ring, such as Phe, Trp, and Tyr have regions with high-value data points on the upper-left area of the MTMs, while in His, a similar region is located on the upper-middle area of the MTM. The MTMs of Pro and His appear to be distinctly different from other MTMs. This could be attributed to the presence of pyrrolidine in Pro and imidazole in His. In order to quantitatively analyze the differences between MTMs of amino acids, a clustered similarity matrix of MTMs was constructed (Figure 4). The similarities of the pairs Cys/Ser, Val/Thr, Asn/Gln, and Asp/Glu were 0.96, 0.81, 0.82, and 0.81, respectively, while Pro and His have no similarity scores larger than 0.61 with respect to other amino acids. Phe, Trp, and Tyr belong to same cluster. The small-sized amino acids, Gly, Ala, Cys, and Ser, with the exception of Val, were clustered into same group. Val has a branched chain, so it may belong to the same group as Thr and Ile. By clustering the MTMs, the 20 amino acids are divided into groups according to their amino acid characteristics (aromatic, acidic, and small-sized side chain among other things). The consistency between the visual interpretation and the clustering of MTM suggests that MTM is a visually interpretive molecular representation. Visual interpretability is important to intuitively understand relationship between input and output data. Thus, it is considered to be one of the major advantages of MTM compared to molecular fingerprints such as Morgan FP and MACCS keys.

### 2.4. Property Prediction Using Molecular Topographic Map

**Figure 5:**
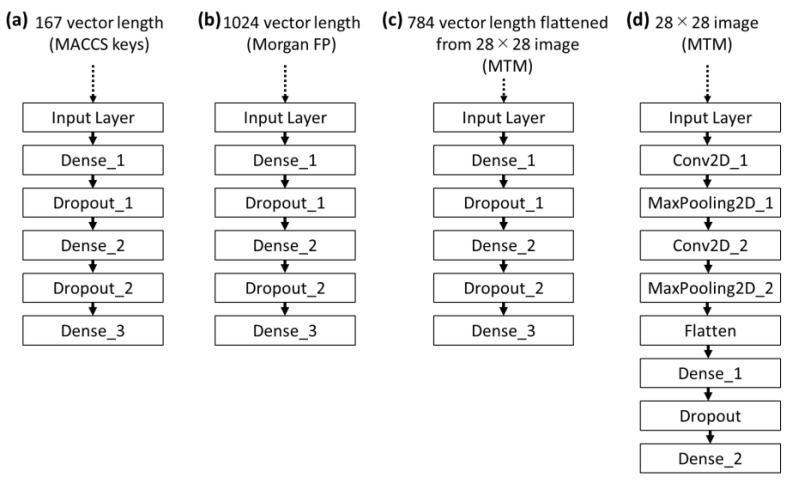
DNN and CNN architectures. (**a**) DNN model using MACCS keys. (**b**) DNN model using Morgan FP. (**c**) DNN model using flattened MTM. (**d**) CNN model using MTM. Dense_X is a fully-connected layer. Dropout_X is a layer where randomly selected neurons are ignored during training. Flatten is a layer that converts a matrix into a single array. Conv2D_X is a convolution layer. MaxPooling2D_X is a pooling layer. X denotes identification number of a layer.

Molecular representations such as fingerprint and descriptors were used as input data to construct predictive models of molecular properties [5,6,7,8,9,10,11,12]. The present study attempts to evaluate predictive models using MTM as input on four public datasets including: (1) a dataset of water solubility for organic small molecules (ESOL), (2) a dataset of hydration free energy of small molecules in water (FreeSolv), (3) a dataset of octanol/water distribution coefficient (logD at pH = 7.4) (Lipophilicity), and (4) a dataset of Caco-2 cell permeability (caco2). The prediction performance is shown in Table 2. CNN is one of the architectures of deep neural network (DNN). In this study, convolutional DNN is referred to as CNN, while non-convolutional DNN is simply referred to as DNN. For MTM, CNN was used to build the predictive models. To confirm an effect of the CNN models, DNN models using flattened MTM as input were built. Furthermore, because generation of MTM is based on Morgan FP algorithm [38,39,40], DNN models using Morgan FP as input were built as baseline predictive models. For comparison with fingerprint, which is different from Morgan FP, DNN models using MACCS keys as input were built. MACCS Keys is a fingerprint that represents the existence of predefined substructures [41]. The four architectures of predictive models are illustrated in Figure 5. CNN model using MTM achieved the best performance on ESOL dataset with an MSE of 0.839, MAE of 0.621, and R^2^ of 0.858, respectively, while the performance of DNN model using flattened MTM (MSE of 0.897, MAE of 0.681, and R^2^ of 0.850) were slightly worse than the CNN model. The DNN model using Morgan FP was the worst model among the four. With the FreeSolv dataset, DNN model using MACCS keys achieved the best performance (MSE of 1.901, MAE of 0.810, and R^2^ of 0.902). With the Lipophilicity dataset, both DNN model using MACCS keys and CNN model using MTM offer comparable performances, but MSE (=0.692) and MAE (=0.610) of the CNN model using MTM was worse than MSE (=0.685) and MAE (=0.605) of DNN model using MACCS keys. The CNN model using MTM gave the best predictions with the caco2 dataset (MSE of 0.179, MAE of 0.321, and R^2^ of 0.718). The CNN model using MTM reached overall high-performance levels and was compatible or superior to DNN models using MACCS keys, flattened MTM, or Morgan FP.

**Figure 6:**
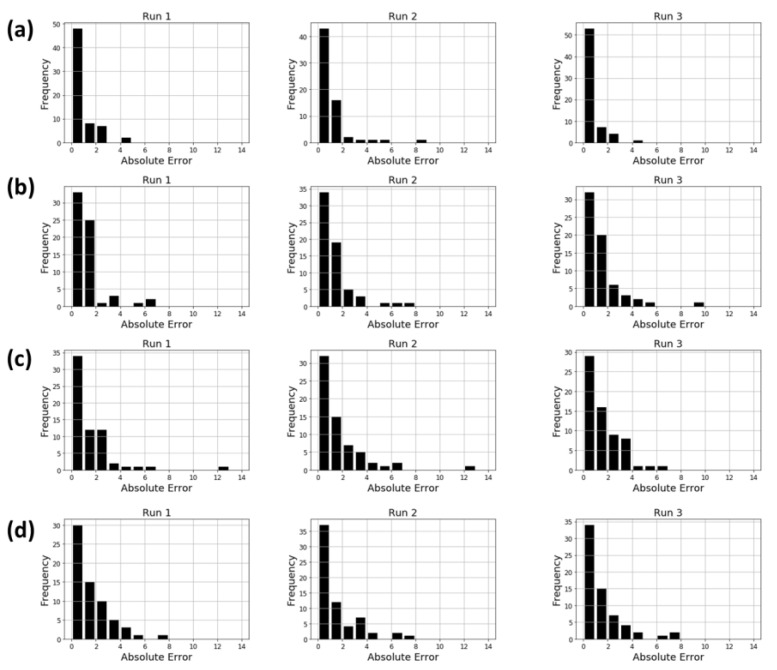
Absolute error distribution of predictive models against FreeSolv dataset. (**a**) DNN model using MACCS keys. (**b**) DNN model using Morgan FP. (**c**) DNN model using flattened MTM. (**d**) CNN model using MTM. Run1, Run2, and Run3 show the absolute error distribution of each of the three independent runs.

The CNN model using MTM had similar or better performance than DNN model using MACCS keys. However, in FreeSolv dataset, DNN model using MACCS keys performed significantly better than the other models. To confirm the details of prediction errors, the histograms of the absolute errors (prediction errors) are plotted in Figure 6. In DNN model using MACCS keys, the number of compounds with absolute error greater than 6 was 0, 1, and 0 for Run 1, Run2, and Run 3, respectively (Figure 6a). On the other hand, CNN model using MTM, the number of compounds with absolute error greater than 6 was 1, 3, and 3 for Run 1, Run2, and Run 3, respectively (Figure 6d). DNN model using Morgan FP and DNN model using flattened MTM showed a similar distribution to CNN model using MTM (Figure 6b,c). The compounds with high absolute error may have a negative impact on prediction performance. MTMs were generated from GTM model built using training data of each property dataset. Thus, in FreeSolv dataset, which is the smallest of the four datasets, the models using MTM may not have been able to produce the high performance, because there is not enough training data to build the GTM. The CNN model using MTM did not perform as well as MACCS keys in FreeSolv dataset, but the results shown in Table 2 suggest that CNN models worked well in processing the MTM and molecular structural information was embedded in the MTM to the same extent as Morgan FP or MACCS keys.

### 2.5. Examples of Relationship between MTM and Its Predicted Molecular Property

**Figure 7:**
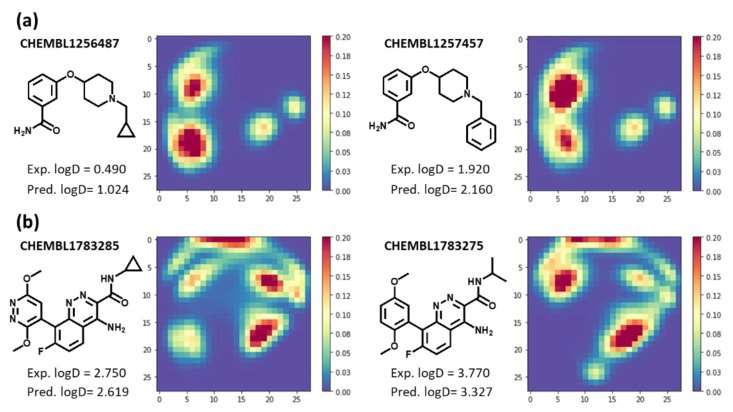
Relationship between MTM and its predicted molecular property (**a**) CHEMBL1256487 and CHEMBL 1257457. (**b**) CHEMBL1783285 and CHEMBL 1783275. Exp. logD means Experimental logD (at pH 7.4) and Pred. logD means Predicted logD (at pH 7.4). The compound identification numbers are ChEMBL [43] identification numbers.

Here, examples of lipophilicity prediction based on CNN model using MTM in the previous section are shown in Figure 7. Predicted logD (at pH 7.4) of CHEMBL1256487 and CHEMBL1257457 are 1.024 and 2.160, respectively. There are high-value data points on lower-left area of MTM of CHEMBL1256487, whereas high-value data points of MTM of CHEMBL1257457 exist on upper-left area (Figure 7a). Cyclopropyl group of CHEMBL1256487 may contribute to the lower-left area of its MTM, whereas benzyl group of CHEMBL1257457 may contribute to the upper-left area of its MTM. The upper-left area of MTM of CHEMBL1257457 seems to contribute mainly to increase predicted logD (at pH 7.4) value. The next example requires a slightly more complicated interpretation (Figure 7b). Predicted logD (at pH 7.4) of CHEMBL1783285 and CHEMBL1783275 are 2.619 and 3.327, respectively. Comparing the two MTMs, high-value data points and middle-value data points exist on upper-right and lower-left area of MTM of CHEMBL1783285, respectively, whereas high-value data points exist on upper-left area of MTM of CHEMBL1783275. The upper-left area of the MTM of CHEMBL1783275 seems to contribute to an increase in predicted logD (at pH 7.4) value as in first example, although the contribution of other areas is not clear from the two MTMs. As shown in Figure 7, unlike molecular fingerprint such as Morgan FP and MACCS keys, MTM is characterized by its ability to visually interpret the relationship between input (MTM) and output (predicted value).

### 2.6. Data Augmentation of Molecular Topographic Map Using MIXUP

**Figure 8:**
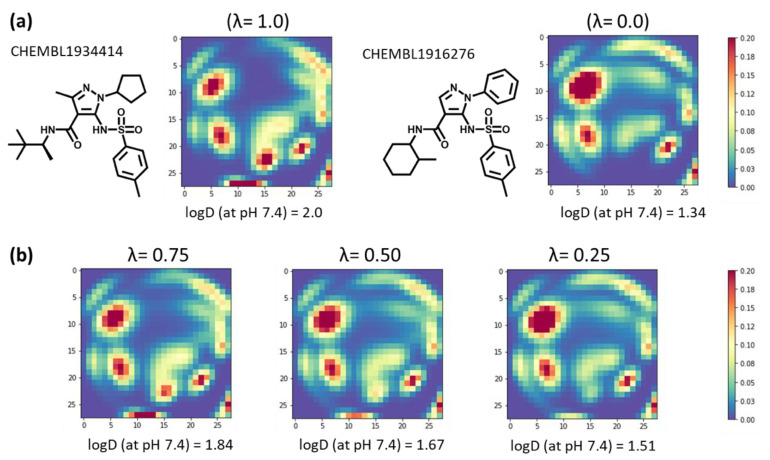
Data augmentation using mixup. λ is the mixing coefficient for both images (MTMs) and their labels (logD (at PH 7.4)). (**a**) Chemical structures and MTMs of CHEMBL1934414 and CHEMBL1916276, respectively. The compound identification numbers are ChEMBL [43] identification numbers. (**b**) Mixed MTMs and their labels with mixing coefficient λ (0.75, 0.50, and 0.25).

Image data augmentation is the most well-known type of data augmentation technique that can be used to expand the size of a dataset by generating transformed versions of images, and improve the performance of predictive models [44]. Here, we used mixup, which is the latest data augmentation technique that linearly interpolates input images and their corresponding labels of random sample pairs. Mixup has demonstrated great effectiveness in image classification [42]. Figure 8 shows, as an exemplary example, data augmentation between two molecules using mixup. The MTMs of CHEMBL1934414 and CHEMBL1916276 with their corresponding labels are shown in Figure 8a. Given two images and their labels, a virtual example is generated as expressed in equation 1, where λ is a mixing coefficient between the two images, and its value ranges from 0 to 1. Figure 8b shows the MTMs and their corresponding labels when the values of λ are 0.75, 0.5, and 0.25, respectively. Three virtually generated MTMs which are linearly interpolated between MTMs of CHEMBL1934414 and CHEMBL1916276 could be used as training data. The effect of data augmentation using mixup on CNN models using MTM are shown in Table 3. Here, λ were values in the range of 0 to 1 and were sampled from the Beta distribution, which is parameterized by the α parameter that controls the shape of the distribution. With the ESOL dataset, no significant effect of data augmentation was observed, although MSE (=0.785) and R^2^ (=0.863) were the best values when the amount of data is doubled and α is 2.0. On the other hand, with other datasets, significant effects of data augmentation were observed. With the FreeSolv dataset, MSE (=3.469), MAE (=1.331), and R^2^ (=0.788) were strikingly improved by increasing the amount of data ten times. Likewise, with the Lipophilicity dataset, the performance improved as the amount of data increased. Where the amount of data is increased ten times and α is 2.0, MSE (=0.607), MAE (=0.576), and R^2^ (=0.597) were the best values. With the caco2 dataset, MSE (=0.151), MAE (=0.285), and R^2^ (=0.736) were improved by increasing the amount of data ten times. Overall, an α of 2.0 was slightly better than an α of 0.2. The results indicate data augmentation using mixup significantly improve the prediction performance of CNN models using MTM, with the exception of ESOL dataset.

## 3. Discussion

The MTM concept was introduced to represent a molecule as an image embedded molecular structure information. In Morgan FP, the bit position of circular substructures around each atom in the fingerprint are determined by hash function [38,39,40]. Thus, there is no relationship between adjacent bits. On the other hand, because MTM is generated based on GTM, adjacent data points on MTM are meant to be similar data points. Hence, the MTM representation is akin to a topographical map, which allows intuitive understanding of the molecular structure and interpret relationship between MTM and its predicted property value.

In terms of performance of molecular property prediction, CNN models using MTM showed better or comparable prediction performance to DNN models using Morgan FP or MACCS keys. However, in FreeSolv dataset, predictive models using MTM did not perform as well as MACCS keys. MTMs are generated from GTM model built using training data of each property dataset. Since FreeSolv dataset is the smallest among the four datasets, there may have been not enough training data to build the GTM model. One of the ways to solve this problem is to construct a generic GTM model using compound data such as ChEMBL [43], instead of using training data for each property dataset. In addition, in order to further improve the prediction accuracy, it may be necessary to consider the size of the radius for generating atomic features set and the matrix size of the MTM. In this study, no comparison has been made on the calculation time among Morgan FP, MACCS keys, or MTM generation. The generation of MTM requires more computational time than that of Morgan FP or MACCS keys because MTM generation requires GTM construction using training data of each property dataset which is a time consuming task. This issue can be solved by constructing generic GTM model in advance.

Furthermore, data augmentation using mixup could improve the prediction performance of CNN models using MTM. One of the advantages of MTM is that it can easily use data augmentation techniques developed in the field of image recognition. In Section 2.5, the relationship between the MTM and its predicted values was interpreted visually, but it may be possible to interpret the relationship by applying explanation methods such as Grad-CAM [45], which is used in the field of image recognition. Grad-CAM provides a coarse localization map highlighting the important regions in the image for predicting a target concept. Therefore, Grad-CAM is expected to be able to identify regions on the MTM that are important for predicting molecular property. To extract chemical information from the important regions of MTM, the relationship between the important regions on the MTM and atoms in the molecular structure needs to be clarified. The relationship is considered to be calculated from atomic features and its responsibility as illustrated in Figure 2. In the future work I intend to develop a method to extract chemical information from the important regions on MTM for predicting molecular property. In this study, although simple and basic CNN architecture was used, the prediction performance of CNN using MTM is expected to be improved by using transfer learning via multiple pretrained networks, such as AlexNet, VGG19, RsNet101, GooLeNet, and Inception-V3 [46]. I believe that MTM embedded molecular structure information can serve as a valuable method of molecular representation for drug discovery. Furthermore, the predictive model using MTM can become a practical tool in drug discovery through combination with state-of-the-art image recognition technologies using deep learning.

## 4. Materials and Methods

### 4.1. Generation of Atomic Features Set

Atomic features set were generated according to the procedure illustrated in Figure 1, which was based on Morgan FP [38,39,40] and neural graph fingerprints [24]. The Morgan FP is a way of encoding the structure of a molecule which is implemented in RDKit [40] as an analogue of extended connectivity fingerprints (ECFP) [39]. In several ligand-based virtual screening studies, ECFP4 showed good performance among different types of 2D fingerprints [47,48]. The number “4” in ECFP4 refers to the diameter of the atom environments. On the other hand, Morgan FP takes radius as parameter. Thus, Morgan FP with radius 2 is equivalent to ECFP4. In the generation of the atomic features set, radius R is set to 2. The atomic features used in the initial assignment to atoms are shown in Table 1. The atomic features consist of atom type, degree, total valence, hybridization, number of hydrogens, formal charge, aromaticity, atoms in rings, and chirality, whose length is 43. All the atomic features were calculated using RDKit [40].

### 4.2. Generation of Molecular Topographic Map

MTM was generated according to the procedure illustrated in Figure 2. GTM models were constructed with runGTM function of ugtm [49], which is a python package that implements GTM [36,37]. In the training of GTM models using the runGTM function, the following parameters were used: k = 28, m = 2, where k is the sqrt of the number of GTM nodes, m is the sqrt of the number of radial basis function centers, and other parameters were set to default. For generating MTMs of amino acids, the GTM model was constructed using 4200 molecules in Lipophilicity dataset as training data obtained from MoleculeNet [50]. In molecular property prediction, GTM model was constructed using training dataset split (80%) from a property dataset. After the construction of the GTM model, the responsibilities of the atomic features were calculated using the transform function of ugtm [49], and MTM is generated by summing up the responsibilities.

### 4.3. Similarity Matrix Using Molecular Topographic Maps

The similarity was calculated by 1/(1 + d), where d was the Euclidean distance between MTM pairs for constructing similarity matrix. Both rows and columns of the similarity matrix were clustered using the clustermap function implemented in seaborn [51], where the metric parameter was set to “correlation”.

### 4.4. Molecular Property Prediction

#### 4.4.1. Property Datasets

To compare property prediction performance of MTM-based, Morgan FP-based, and MACCS keys-based models, three datasets, including ESOL, FreeSolv, and Lipophilicity, were obtained from MoleculeNet [50] and caco2 was obtained from literature [52]. ESOL is a dataset of water solubility for organic small molecules, and is composed of 1128 molecules. FreeSolv is a dataset of hydration free energy of small molecules in water, and it contains 642 molecules. Lipophilicity is a dataset of octanol/water distribution coefficient (logD at pH = 7.4) containing 4200 molecules. caco2 is a dataset of Caco-2 cell permeability containing 1272 molecules. The four datasets were used for the regression tasks.

#### 4.4.2. Molecular Representation

Morgan FP was calculated with a radius of 2 and a length of 1024 bits using RDKit [40] for all molecules. MACCS keys consists of 167-bit-long fingerprint was calculated using RDKit [40]. MTMs of molecules in ESOL dataset were calculated using a GTM model constructed using a training dataset split (80%) from ESOL dataset. In the same way, MTMs of molecules in the FreeSolv, Lipophilicity, and caco2 dataset were calculated.

#### 4.4.3. Calculation Protocol

Three DNN and one CNN models were built to predict molecular property from chemical structures (Figure 5). These models were implemented with Tensorflow [53] and Keras [54]. In order to evaluate the DNN models, three independent runs based on different random seeds of data split into training, validation, and test sets at the ratio of 8:1:1, were performed against each property dataset (ESOL, FreeSolv, Lipophilicity, and caco2). For each independent run, hyperparameters of the models were optimized using optuna, which is an automatic hyperparameter optimization software framework [55]. In the DNN models illustrated in Figure 5a–c, the following hyperparameters were optimized to minimize mean squared error of the validation set: number of units of “Dense_1” (256, 512, 768, 1024), dropout rates of “Dropout_1” (0 to 0.9 every 0.1), number of units of “Dense_2” (256, 512, 768, 1024), and dropout rates of “Dropout_2” (0 to 0.9 every 0.1). The following hyperparameters were fixed: activation functions (ReLU) of ”Dense_1” and “Dense_2”, batch size (16), epochs (100), and optimizer (Adam). In the CNN model illustrated in Figure 5d, the following hyperparameters were optimized to minimize mean squared error of the validation set: number of filters of “Conv2D_1” (16 to 96 every 16), number of filters of “Conv2D_2” (16 to 96 every 16), number of units of “Dense_1” (256, 512, 768, 1024), and dropout rates of “Dropout” (0 to 0.9 every 0.1). The following hyperparameters were fixed: kernel size of “Conv2D_1” and “Conv2D_2” ((5,5)), pooling window size of “MaxPooling2D_1” and “MaxPooling2D_1” ((2,2)), activation functions (ReLU) of “Conv2D_1”, “Conv2D_2”, and “Dense_1”, batch size (16), epochs (100), and optimizer (Adam). Finally, These models were constructed using the optimized parameters, and evaluated by average performance against test sets over three runs. Different measures were used to evaluate the performance including mean squared error (MSE), mean absolute error (MAE), and square of correlation coefficient (R^2^).

#### 4.4.4. Data Augmentation Using Mixup

Mixup is one of the data augmentation techniques being used to generate new training examples by linearly interpolating input images and the corresponding labels [42]. Given randomly selected two images and their corresponding labels: $\left(x_{i},y_{i}\right)$, $\left(x_{j},y_{j}\right)$ (*x* is an image and *y* is its one-hot encoding label), a synthetic training example $\left(\hat{x},\hat{y}\right)$ is generated as:

(1) $$\begin{array}{c} \hat{x}=\lambda x_{i}+\left(1-\lambda \right)x_{j}\\ \hat{y}=\lambda y_{i}+\left(1-\lambda \right)y_{j} \end{array}$$

where *λ*~Beta(*α*, *α*) for each pair of examples, with an *α* hyperparameter.

In this study, *y* is a real number that represents molecular property instead of one-hot encoding label.

## Figures and Tables

**Table 1:** Forty-three atomic features.

ID	Description		ID	Description	
1	H	Atom type as a one-hot vector	25	S	Hybridization of an atom as one-hot vector
2	C		26	SP	
3	N		27	SP2	
4	O		28	SP3	
5	S		29	SP3D	
6	P		30	SP3D2	
7	F		31	0	Number of hydrogens on an atom as one-hot vector
8	Cl		32	1	
9	Br		33	2	
10	I		34	3	
11	0	Degree of an atom as one-hot vector, which defined to be its number of directly-bonded neighbors.	35	4	
12	1		36	−1	Formal charge of an atom as one-hot vector
13	2		37	0	
14	3		38	1	
15	4		39	Aromatic	Is aromatic
16	5		40	Ring	Is in ring
17	6		41	R	Chirality of an atom as one-hot vector
18	0	Total valence of an atom as one-hot vector	42	S	
19	1		43	Non-chiral	
20	2				
21	3				
22	4				
23	5				
24	6				

**Table 2:** Performance comparison of the four datasets for four models.

Dataset	No.	Molecular Representation	Model	MSE		MAE		R^2^	
ESOL	1128	MACCS keys	DNN	1.202	(±0.187)	0.789	(±0.048)	0.781	(±0.011)
		Morgan FP	DNN	1.592	(±0.108)	0.942	(±0.041)	0.705	(±0.018)
		flattened MTM	DNN	0.897	(±0.178)	0.681	(±0.056)	0.850	(±0.028)
		MTM	CNN	0.839	(±0.166)	0.621	(±0.059)	0.858	(±0.021)
FreeSolv	642	MACCS keys	DNN	1.901	(±0.834)	0.810	(±0.149)	0.902	(±0.035)
		Morgan FP	DNN	5.007	(±1.495)	1.402	(±0.120)	0.741	(±0.029)
		flattened MTM	DNN	7.114	(±2.293)	1.701	(±0.109)	0.696	(±0.082)
		MTM	CNN	4.864	(±0.180)	1.531	(±0.030)	0.727	(±0.070)
Lipophilicity	4200	MACCS keys	DNN	0.685	(±0.024)	0.605	(±0.019)	0.551	(±0.012)
		Morgan FP	DNN	0.705	(±0.049)	0.623	(±0.025)	0.539	(±0.005)
		flattened MTM	DNN	0.707	(±0.040)	0.626	(±0.016)	0.537	(±0.026)
		MTM	CNN	0.692	(±0.066)	0.610	(±0.029)	0.554	(±0.031)
caco2	1272	MACCS keys	DNN	0.196	(±0.040)	0.351	(±0.050)	0.711	(±0.021)
		Morgan FP	DNN	0.210	(±0.056)	0.337	(±0.048)	0.684	(±0.059)
		flattened MTM	DNN	0.196	(±0.012)	0.336	(±0.012)	0.655	(±0.051)
		MTM	CNN	0.179	(±0.021)	0.321	(±0.024)	0.718	(±0.024)

Mean (±standard deviation) values are reported for multiple measures (MSE, MAE, R^2^).

**Table 3:** Effect of mixup on performance of the four datasets using CNN model using MTM.

Dataset	No.	α	MSE		MAE		R^2^	
ESOL	1128	-	0.839	(±0.166)	0.621	(±0.059)	0.858	(±0.021)
	1128 × 2	0.2	0.833	(±0.125)	0.643	(±0.067)	0.848	(±0.020)
	1128 × 2	2.0	0.785	(±0.086)	0.649	(±0.034)	0.863	(±0.017)
	1128 ×10	0.2	0.890	(±0.153)	0.662	(±0.069)	0.839	(±0.018)
	1128 × 10	2.0	0.851	(±0.149)	0.674	(±0.049)	0.851	(±0.033)
FreeSolv	642	-	4.864	(±0.18)	1.531	(±0.030)	0.727	(±0.070)
	642 × 2	0.2	4.215	(±0.396)	1.414	(±0.035)	0.755	(±0.065)
	642 × 2	2.0	4.274	(±0.467)	1.469	(±0.089)	0.746	(±0.102)
	642 × 10	0.2	3.642	(±0.414)	1.331	(±0.161)	0.770	(±0.091)
	642 × 10	2.0	3.469	(±0.400)	1.332	(±0.061)	0.788	(±0.081)
Lipophilicity	4200	-	0.692	(±0.066)	0.610	(±0.029)	0.554	(±0.031)
	4200 × 2	0.2	0.642	(±0.050)	0.588	(±0.025)	0.576	(±0.025)
	4200 × 2	2.0	0.627	(±0.075)	0.588	(±0.035)	0.586	(±0.033)
	4200 × 10	0.2	0.646	(±0.076)	0.595	(±0.032)	0.572	(±0.037)
	4200 × 10	2.0	0.607	(±0.054)	0.576	(±0.021)	0.597	(±0.027)
caco2	1272	-	0.179	(±0.021)	0.321	(±0.024)	0.718	(±0.024)
	1271 × 2	0.2	0.161	(±0.005)	0.303	(±0.007)	0.722	(±0.023)
	1272 × 2	2.0	0.189	(±0.032)	0.324	(±0.022)	0.696	(±0.020)
	1272 × 10	0.2	0.151	(±0.011)	0.288	(±0.011)	0.736	(±0.017)
	1272 × 10	2.0	0.151	(±0.009)	0.285	(±0.007)	0.732	(±0.010)

Mean (±standard deviation) values are reported for multiple measures (MSE, MAE, R^2^).

## Data Availability

Not applicable.
